# The Evolution of Standards in Dental Education

**Published:** 1904-09

**Authors:** Charles C. Chittenden

**Affiliations:** Madison, Wis.


### THE EVOLUTION OF STANDARDS IN DENTAL EDUCATION.[1]

BY CHARLES C. CHITTENDEN, D.D.S., MADISON, WIS.

In a paper read before this Section one year ago I reviewed the various steps by which dental educational standards had been slowly and laboriously advanced from a mere shadowy pretence of individual schools to the establishment by the National Association of Dental Faculties of a minimum educational requirement for admission to all schools, which was equivalent to two full years of high school, and also the adoption of a full course of instruction for graduation of four years of seven months each.

The four years' course was proposed by resolution in that body in 1899. Action was laid over for one year, and at Mil-

---

[1] Read at the fifty-fifth annual session of the American Medical Association, in the Section on Stomatology, Atlantic City, June 7 to 10, 1904.

waukee, in 1900, the Faculties' Association deliberately and after careful consideration decided on its adoption, setting forward the time for its inauguration to the autumn of 1903, thus reserving three years more in which it would be possible to change their decision in case it should develop that the time was not ripe for such advance. Thus at the annual meeting of 1903, held at Asheville, N. C., the subject came up for final irretrievable action, which was precipitated by Harvard University Dental Department.

That school informed the Association that it would "not at present extend its course to four years," assigning as its reason that it was engaged in contemplating the raising of its educational requirements for entrance at some time in the future.

A special committee had the matter under advisement for three days as to whether the Association should "concede that Harvard is entitled to one year less time than other schools having an equal standard of admission, with the exception, it may be, of analytical chemistry and physics." The committee reported recommending that Harvard could not be granted such concession, and after a free discussion the report was unanimously adopted on a roll-call of colleges. Harvard then resigned its association membership, and the resignation was promptly accepted. In all this the National Association of Dental Faculties deliberately, unanimously, and finally placed itself on record as standing firmly for the four years' course.

Simultaneously at Asheville an attempt was made in the National Association of Dental Examiners to have rescinded the following standing resolution, adopted at Milwaukee in 1900 as a treaty of peace and good faith between the colleges and the examiners,—viz.:

*Resolved,* That no college not an acceptable member of the National Association of Dental Faculties be placed on or continued on the recommended list of this association.

This attempt to rescind failed, and the two associations adjourned in full accord and with the mutual conviction that the four years' course was established for all time, and that the two associations would stand firmly together for its maintenance as a criterion of the reputability of dental colleges.

Thus it transpired that every dental college in the Faculties'

Association, and, in fact, every dental college in the United States, save one, opened up the school year of 1903-04 with the four years' course inaugurated and advertised to the world as the established standard deemed necessary by the educators themselves for the proper preparation of the dental student for graduation and practice. It was a brave move,—a grand achievement,—and the profession at large gave a great sigh of satisfaction that the schools had made this advance step towards removing the stigma which had been cast in foreign countries on the educational methods and standards of the schools of the United States.

It seemed that there remained only the necessity to secure the further requirement of high-school graduation for matriculation (which step had been practically promised by most of our universities and actually inaugurated in some of them) to place this country in the forefront of the nations, with practically no peer. The dream of raising up a generation of educated, scientific professional gentlemen to serve as the standard bearers of dentistry for the near future seemed about to be realized.

The school year for 1903-04 opened with the new order of things, but to the great disappointment of many deans, whose freshman classes were much smaller than the year previous. This condition was largely accounted for by the fact that prospective students had hastened to take advantage of a last chance to graduate in a three years' course and had flocked to the schools the year before in large numbers. This fact also made the first four years' freshman classes seem abnormally small. Of course, the income to the schools was correspondingly small. To the older-established and the endowed schools and departments of universities this did not so much matter, as the check was, of course, only temporary. But the smaller proprietary schools at once took alarm at the decrease in income, and without regard, it would seem, to what effect such a course would have on their standing and reputability in the eyes of the public, began, within a month after the opening of the school year, to devise some plan for inducing the National Association of Dental Faculties to return to the old course of three years for graduation. An effort (which finally proved successful) was made to hold an extra meeting of the Association at Buffalo during the Christmas holidays to act on such a proposition. At this meeting it was held and ruled by the president that no action could be taken or business transacted until the next regular annual meeting, but much time was spent in discussing the situation. Sentiment was divided about as follows: One factor, consisting of about one-third of the Association membership and representing almost exclusively the proprietary schools struggling for existence, were avowedly for an unequivocal return to the three years' course of seven, or even six, months each. Another smaller fraction favored a high-school diploma for matriculation and a three years' course of nine months for graduation. And, lastly, those schools having adequate financial foundation on which to bank in conducting their work on any reasonably advanced standards, those schools representing about one-half the membership in the Association, stood and pleaded for professional honor and professional unity—stood firmly for no retrograde of standards and requirements. When the meeting adjourned it seemed reasonable to believe that no further attempt at lowering standards would be made.

When the national body adjourned at Asheville in 1903 it was with the general understanding that the next annual session would be held at St. Louis at about the same time in August, 1904, as would be held the International Dental Congress,—that is, the latter part of that month.

It has been the rule of the schools to issue their annual announcements for the coming school year course some time in June, as the business for the coming year begins immediately after the old term closes. Had the National Association of Dental Faculties held to the plan laid out at Asheville as to the time of the annual meeting, these announcements would all have been issued on the basis of a four years' course before the meeting could occur, and a change in the course would have been practically impossible; but for reasons best known to themselves the majority of members have recently decided to hold the meeting in the city of Washington, June 9. Such a change in programme is so unusual that it is but natural to seek a reasonable explanation. At this early date the annual announcements will not have been issued, and it would be entirely reasonable to infer that another attempt may be made to lower standards. Should such an attempt prove successful, there are a great many things likely to happen. It is not conceivable that the endowed and independent schools will tamely submit (even should they be in the minority) to a retrograde educational movement. They could in no sense afford it, nor would it be in accord with any of their traditions. They have for years submitted to being held back by the inability of the weaker schools to move forward rapidly on account of their financial handicap. It is but reasonable to expect that the Faculties' Association would either go to pieces or become divided into two separate organizations with separate standards.

The question at once presents: What would be the attitude of the legally appointed examiners in the States which hold their boards responsible as to the judging of the reputability and standing of the colleges from which their applicants for license have graduated?

What would be the attitude of the profession at large? Will the men who are out in the open, bearing the heat and burden of the day before the public, tamely submit to the open discredit that would be cast on their chosen calling by a national lowering of its educational standards?

It seems impossible that such a calamity as a division of college forces on this question could occur, and still more impossible that a decided retrograde movement can possibly receive the sanction of a majority of the schools. But should this happen, it is safe to predict that the rank and file of dentists will put their stamp of disapproval on such a transaction in no uncertain manner. The prediction is reasonable that schools of the trimming and retrograde type would be allowed to fall back out of sight and be forgotten in the onward march of the development and evolution of dental educational standards.

